# The role of the tumour microenvironment in lung cancer and its therapeutic implications

**DOI:** 10.1007/s12032-025-02765-7

**Published:** 2025-05-23

**Authors:** Devindi Thathsara Edirisinghe, Jasleen Kaur, Yue Qi Lee, Huey Xin Lim, Sharis Wan Ting Lo, Sri Vishupriyaa, Ee Wern Tan, Rebecca Shin Yee Wong, Bey Hing Goh

**Affiliations:** 1 Department of Medical Education, Sir Jeffrey Cheah Sunway Medical School, Faculty of Medical and Life Sciences, Sunway University, No. 5 Jalan Universiti, 47500 Petaling Jaya, Selangor Darul Ehsan Malaysia; 2 Sunway Biofunctional Molecules Discovery Centre, Faculty of Medical and Life Sciences, Sunway University, No. 5 Jalan Universiti, 47500 Petaling Jaya, Selangor Darul Ehsan Malaysia; 3 Faculty of Health, Australian Research Centre in Complementary and Integrative Medicine, University of Technology Sydney, Ultimo, Australia; 4 Graduate Institute of Cancer Biology and Drug Discovery, College of Medical Science and Technology, Taipei Medical University, Taipei, Taiwan

**Keywords:** Tumour microenvironment, Therapeutic implications, Lung cancer, NF-κB pathway, STAT3 pathway

## Abstract

Lung cancer is the leading cause of cancer-related deaths globally, with tumour growth, invasion, and treatment response heavily influenced by the tumour microenvironment (TME). The TME promotes tumour progression by creating an immunosuppressive environment that hampers the body’s antitumour immune response, primarily through the Nuclear Factor Kappa B (NF-κB) and Signal Transducer and Activator of Transcription 3 (STAT3) pathways. These pathways contribute to chronic inflammation, immune evasion, and angiogenesis. Targeting the TME and its signalling pathways has shown potential to enhance treatment efficacy. STAT3, a key transcription factor in lung cancer, drives tumour growth and immune suppression via the mTOR and JAK pathways. Inhibiting these pathways can block STAT3 and slow cancer progression. Promising results have been observed with mTOR inhibitors like CC-115 and Vistusertib, especially when combined with immune checkpoint inhibitors, and with JAK inhibitors such as Ruxolitinib, AZD4205, and Filgotinib. These strategies represent a promising direction for lung cancer therapy. This review explores the intricate relationship between the TME and lung cancer, focussing on novel therapeutic approaches that target immune cells, signalling molecules, and fibroblasts within the TME to improve patient outcomes.

**Graphical abstract:**

Overview of the lung TME, illustrating key cellular components, signalling pathways, and their roles in tumour proliferation, metastasis, immune evasion, and angiogenesis. 
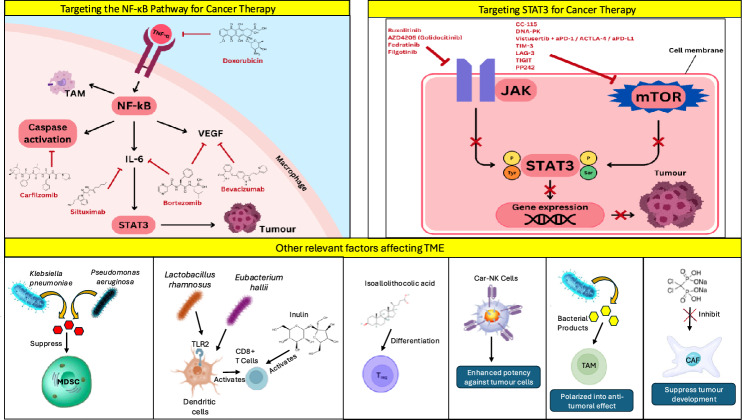

## Introduction

Bronchogenic carcinoma, also known as lung cancer, occurs due to the development of tumours within the bronchial airways or the parenchyma and has the highest mortality rate for cancer-related deaths globally [1]. The two main types of lung cancer are Non-Small Cell Lung Cancer (NSCLC), accounting for 85% of cases, and Small Cell Lung Cancer (SCLC), accounting for 15% of cases, despite its extremely aggressive nature. The late diagnosis of lung cancer at advanced stages leads to the limited available treatment options. Therefore, early detection along with improved therapeutic strategies is extremely crucial to increase the patient survival rates [2].

In the past decade, the considerable influence of TME on the onset and advancement of primary de novo lung carcinoma has been gaining substantial recognition. The TME is a complex microenvironment that plays a key role in tumour growth, metastasis, and therapeutic intervention response. TME consists of cellular and non-cellular factors, such as the stromal cells, immune cells, signalling molecules and the extracellular matrix, which interact with one another thereby regulating the tumour behaviour. Recent research has revealed that the composition of immune cells within the tumour environment influences the prognosis of lung cancer in patients [3].

In inflammatory conditions such as chronic obstructive pulmonary disease (COPD), the lung TME exhibits features that may facilitate carcinogenesis. The human lung adenocarcinoma displays a range of subtypes, where each are defined by distinct cellular and mutational heterogeneity. Notably, this heterogeneity is not limited to the tumour epithelial cells and could extend beyond to encompass the TME, which includes the vasculature, cancer-associated fibroblast (CAFs), extracellular matrix (ECM), and infiltrating immune cells. In NSCLC, the degree of immune cell infiltration is dependent on the disease stage, suggesting that the lung TME influences carcinogenesis and may serve as a prognostic factor. Consequently, specific TME profiles are under investigation to be used as potential biomarkers for assessing disease stage and subtype along with predicting clinical outcomes and guiding therapeutic strategies [4].

The TME also consists of a vast array of immune cells such as myeloid-derived suppressor cells, tolerogenic dendritic cells, tumour-associated macrophages and regulatory T cells. The accumulation of these cells results in an immunosuppressive environment thereby, hindering the antitumour immune response of the body. This is one of the key reasons why targeting the TME is crucial for improving treatments for lung cancer [5]. Hence, this literature review aims to explain the extensive roles of the TME in lung cancer, primarily focussing on the immunosuppressive cells, cancer-related fibroblasts and identifying therapeutic strategies that could enhance treatment efficacy and patient outcomes.

## TME and its mechanisms

### TME

The metabolic exchange of substances (such as nutrients and oxygen) is not only essential for the survival of normal lung cells but is also crucial in maintaining the lung TME to enhance malignant cell growth. The TME plays a crucial role in the growth and metastasis of lung cancer. It consists of a heterogenous mixture of cell populations, including cancer cells, stromal cells, blood vessels, immune cells, signalling mediators and extracellular matrix proteins. The environment of chronic inflammation in TME contributes to altered immune cell differentiation and hinders antitumour activity, ultimately increasing tumour evasion [6]. Therefore, the TME can potentially be used in prognostics of NSCLC for determining the stage, clinical outcomes, and response to therapy [4].

In addition, chronic inflammation within the TME is driven by various cytokines, chemokines, and other signalling molecules that shape the tumour microenvironment and promote tumour progression. These factors create an immune-evasive environment, facilitating cancer cell survival, invasion, and metastasis [7]. An example of a cytokine involved in the TME and implicated in the progression of lung cancer is interleukin 8 (IL-8). Research has shown that CAFs are activated by lactate produced by tumour cells, leading to increased secretion of IL-8 [8].

Besides chronic inflammation, vessel co-option also plays a role in tumourigenesis and responses to therapies. Vessel co-option refers to a non-angiogenic process where cancer cells utilize pre-existing blood vessels for vascularization instead of inducing new vessel formation. It has been observed across various tumour types, including primary and metastatic lung cancer. Importantly, vessel co-option is now implicated as a major mechanism mediating resistance to conventional anti-angiogenic drugs, potentially explaining their limited efficacy in treating advanced-stage/metastatic lung cancer, in adjuvant settings, and for primary lung tumours. Vessel co-option may influence responses to anti-angiogenic and immunotherapies in lung cancer and suggests a need for therapies targeting both angiogenesis and vessel co-option [9].

On the other hand, serglycin (SRGN), a factor within the lung TME secreted by both tumour and stromal cells, promotes NSCLC aggressiveness. This occurs through its interaction with the CD44 receptor on NSCLC cells, leading to increased cell migration, invasion, and colonization. SRGN also induces stem-like properties, enhances resistance to chemotherapy and anoikis, and promotes epithelial-mesenchymal transition (EMT). Notably, increased SRGN expression is associated with poorer prognosis in lung adenocarcinoma, highlighting its significant role in driving lung cancer progression [10].

### NF-κB and STAT3 pathways involved in the lung TME

Cytokines are crucial components that contribute to chronic inflammation, which is linked to cancer. It also enhances the formation of malignant cells, promotion of metastasis and immune cell evasion. There are several inflammatory pathways involved in the maintenance of TME in lung cancer. Two of the main pathways are NF-κB and STAT3, which can be activated by cytokines, leading to cellular gene damage and mutations, ultimately resulting in tumour development. Moreover, these pathways also increase inflammatory factor production to upkeep the TME and contribute to inflammation and the tumour cycle [11]. The resulting production of inflammatory factors creates a vicious cycle, supplying signals for inflammatory pathways, and therefore further production of cytokines. Some of the inflammatory factors involved in lung cancer are IL-6, IL-11, IL-12 and TNF-α.

The overactivation of NF-κB pathway causes increased cell survival, vascularisation, and cell invasion, all of which are factors that lead to development of cancer. Additionally, NF-κB inhibits apoptosis through several mechanisms, including the degradation or inhibition of caspases and the induction of inhibitors of apoptosis (IAPs) [12]. Hence, as NF-κB has strong correlations to the inflammation of tumours, studies have showed that as NF-κB can function as a target for therapeutic strategies [12]. The NF-κB pathway can be activated by cytokines such as TNF-α, IFN-γ and IL-17, as well as oncogenes and tumour suppressors. After stimulation, the IκB kinase complex (IKK) phosphorylates IκB proteins, which are bound to NF-κB. This results in the degradation of IκB proteins by proteasomes, causing the release of NF-κB proteins [13]. The signal transduction cascade ultimately enables the translocation of NF-κB complex into the nucleus, activating transcription of various inflammatory genes [14]. Hence, the activation of NF-κB pathway is often present in the TME and cancer cells in most solid tumours.

Signal transducer and activator of transcription 3 (STAT3) is a transcription factor that is constitutively activated in cancer cells and functions as an oncogene essential for carcinogenesis and tumour development. It regulates processes such as tumour cell survival, proliferation, invasion, and inflammation [15]. STAT3 promotes angiogenesis through hypoxia-inducible factors (HIF) and vascular endothelial growth factor A (VEGFA). The knockdown of STAT3 has been shown to completely ablate the expression of hypoxia-inducible factor-1 α (HIF-1α), hypoxia-inducible factor-2 α (HIF-2α) and VEGFA, all of which are critical for angiogenesis [16]. STAT3 is a downstream target of mechanistic target of rapamycin complex 1 (mTORC1) and is activated by cytokines or growth factors binding to cell surface receptors. Full activation of STAT3 requires phosphorylation at two sites: tyrosine 705 (Tyr705) and serine 727 (Ser727). When interleukin 6 (IL-6) binds to its cell surface receptor, it recruits and activate Janus kinase (JAK) family members, which phosphorylates STAT3 at Tyr705, facilitating nuclear translocation and enhancing cytokine-mediated gene expression. Additionally, mTORC1 directly phosphorylates STAT3 at Ser 727, driving cancer cell proliferation and the formation of large cancer cell clusters.

**Fig. 1:**
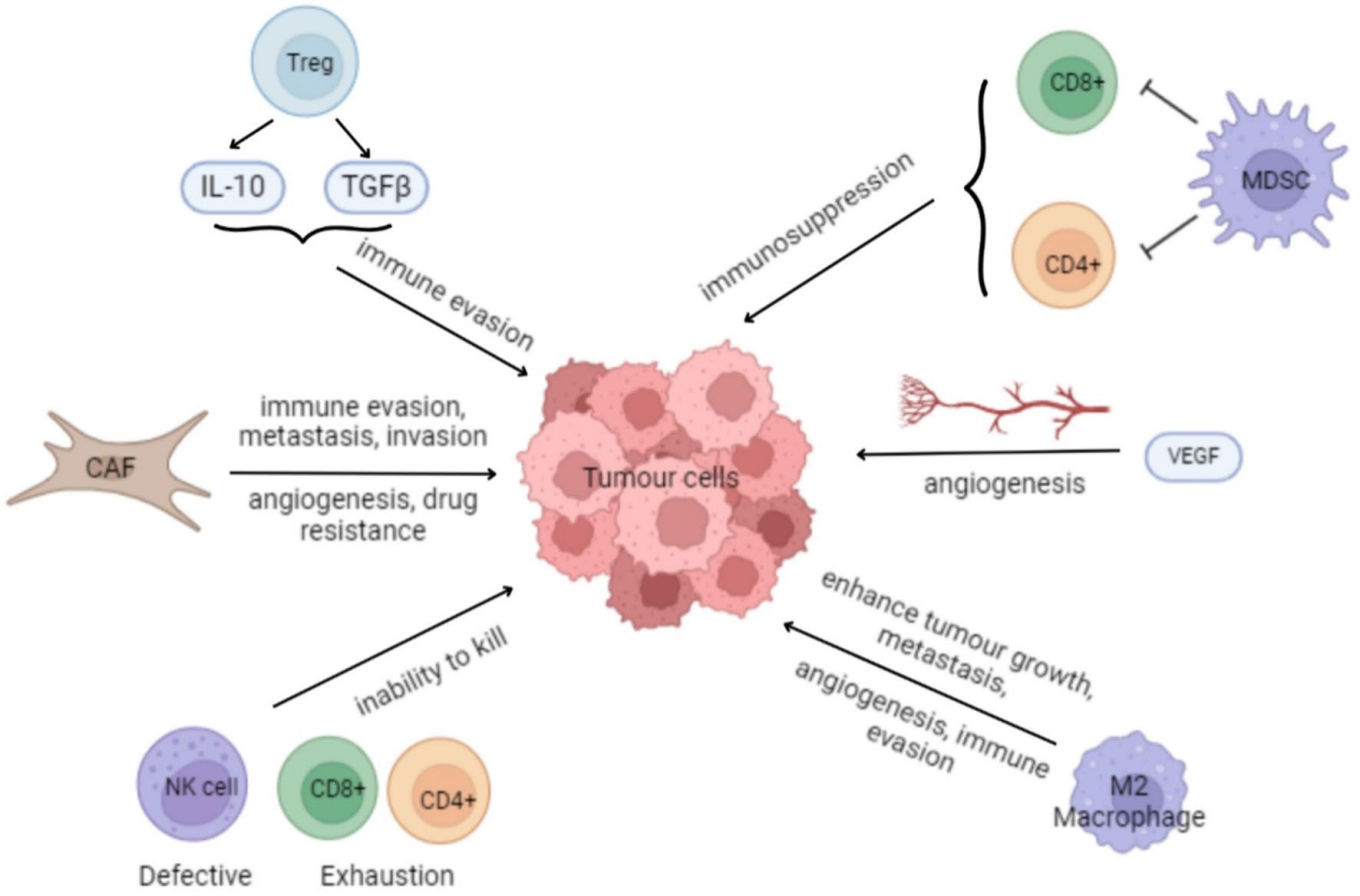
Roles of different cells in the TME of lung cancer

Importantly, aberrant STAT3 signalling through pathways such as VEGFA not only drives tumour growth but also contributes to drug resistance by promoting a tumour-supportive microenvironment, enhancing cancer cell survival, and impairing therapeutic efficacy. Drug resistance remains a major challenge in cancer therapy, often arising from both intrinsic and acquired mechanisms, including genetic mutations, epigenetic alterations, cancer stem cell plasticity, and exosome-mediated communication, all of which contribute to treatment failure and disease progression [17]. Taken together, the roles of cytokines, NF-κB, and STAT3 in cancer development and TME regulation is tabulated in Table 1 and illustrated in Fig. 1 below.

**Table 1:** Roles of cytokines, NF-κB, and STAT3 in cancer development and TME regulation

Cytokine/Pathyway	Key findings	Reference
Cytokines	• Promote chronic inflammation and tumour growth • Enhance metastasis and immune evasion • Activate NF-κB and STAT3 pathways • Sustain TME via IL-6, IL-11, IL-12, TNF-α	[11]
NF-κB	• Increases cell survival, angiogenesis, invasion • Inhibits apoptosis (blocks caspases, induces IAPs) • Activated by TNF-α, IFN-γ, IL-17 • IKK degrades IκB, freeing NF-κB to trigger inflammatory genes	[12–14]
STAT3	• Drives tumour growth, angiogenesis (HIF/VEGFA) • Activated by IL-6/JAK (Tyr705) and mTORC1 (Ser727) • Knockdown suppresses HIF-1α, HIF-2α, VEGFA • Promotes cancer cell proliferation and clustering	[15, 16]

### Cells involved in the lung TME

The overall effects of various cells within the TME are summarized in Fig. 1. Tumour-associated macrophages (TAMs) are key contributors to the TME, secreting inflammatory mediators that drive chronic inflammation. TAMs are broadly categorized into two types: M1 and M2 macrophages. M1 macrophages primarily produce IL-12 and IL-23, which are involved in pathogen destruction and tumour cell cytotoxicity. Conversely, M2 macrophages promote tumour stroma formation, tumour growth, metastasis, and angiogenesis. In the lung cancer TME, signalling pathways lead to an imbalance favouring a higher proportion of M2 macrophages over M1, thus promoting tumour progression and immune evasion [18]. Additionally, macrophage activity is modulated by tumour cell transcription factors, such as NF-κB and STAT3. The interplay between these factors fosters a microenvironment conducive to cancer cell proliferation, inhibition of apoptosis, angiogenesis, extracellular matrix remodelling and metastasis.

Myeloid-derived suppressor cells (MDSCs) are key regulators within the TME of lung cancer. MDSCs are immune precursor cells that contribute to immunosuppression and facilitate immune evasion by targeting the T cells. Additionally, MDSCs produce vascular endothelial growth factor (VEGF), which enhances tumour growth [19]. Overexpression of VEGF leads to the formation of new blood vessels, supplying oxygen and nutrients to tumour cells, thereby promoting tumour invasion and metastasis [20]. Studies have demonstrated a strong correlation between peripheral blood MDSC levels and tumour stage and metastasis, indicating that reducing MDSCs levels could inhibit tumour progression and metastasis [19]. Elevated levels of MDSCs within the TME further enhance immunosuppression.

The main immune cells that combat cancer are CD8^+^ T cells and CD4^+^ T cells. CD8^+^ T cells kill cancer cells by migrating into the TME, differentiating into effector CD8^+^ T cells, and then into cytotoxic and memory CD8^+^ T cells. To accomplish this, external and internal transcription factors regulate the cell’s surface receptors, induces multiplication, and produces effectors to kill the cancer cells within the TME. However, the normal immune functions of lung cancer patients may be impaired, as dysfunction of the CD8^+^ T cells can occur [21]. Conversely, accumulation of CD4^+^ T cells such as Th2 cells and Tregs can be observed in the stroma and epithelium in lung cancer.

Regulatory T-lymphocytes (Tregs) are a subset of CD4^+^ T cells that contribute to an immunosuppressive TME by secreting interleukin 10 (IL-10) and transforming growth factor β (TGFβ). TGFβ promotes the transcription of Forkhead box P3 (FOXP3), driving the differentiation of naïve CD4 T cells into Tregs [22]. Elevated levels of tumour-infiltrating Tregs are associated with poorer overall survival in lung cancer [23]. In contrast to conventional CD4^+^ T cells that enhance immune responses, Tregs maintain immune homeostasis and self-tolerance by suppressing the activity of other immune cells, thereby preventing autoimmune reactions. The IL-10 and TGFβ produced by Tregs inhibit the activation of effector T cells and other immune cells, promoting tumour immune evasion.

CAFs are the predominant cell type in lung cancer. Unlike normal fibroblasts which are typically inactive and supports the extracellular matrix (ECM) during wound healing, CAF undergoes irreversible activation. Moreover, they also have increased migratory and proliferative abilities and secrete various molecules. CAFs play crucial roles in immune evasion, metastasis, invasion, angiogenesis, and drug resistance, thus enhancing the TME [24]. Furthermore, CAFs are involved in stimulating inflammation which facilitates tumour growth. As CAFs can secrete cytokines and chemokines, these molecules recruit and activate immune cells, and their reciprocal interactions contribute to the TME. In lung cancer, CAFs produce an excess of tryptophan 2–3-dioxygenase (TDO2) which inhibits dendritic cell differentiation [25]. Recent studies have identified subtypes of CAFs, such as myofibroblastic CAFs (myCAFs), which are key in promoting tumour growth and therapy resistance. Targeting myCAFs or combining strategies with immunotherapy may enhance treatment outcomes [26].

**Fig. 2:**
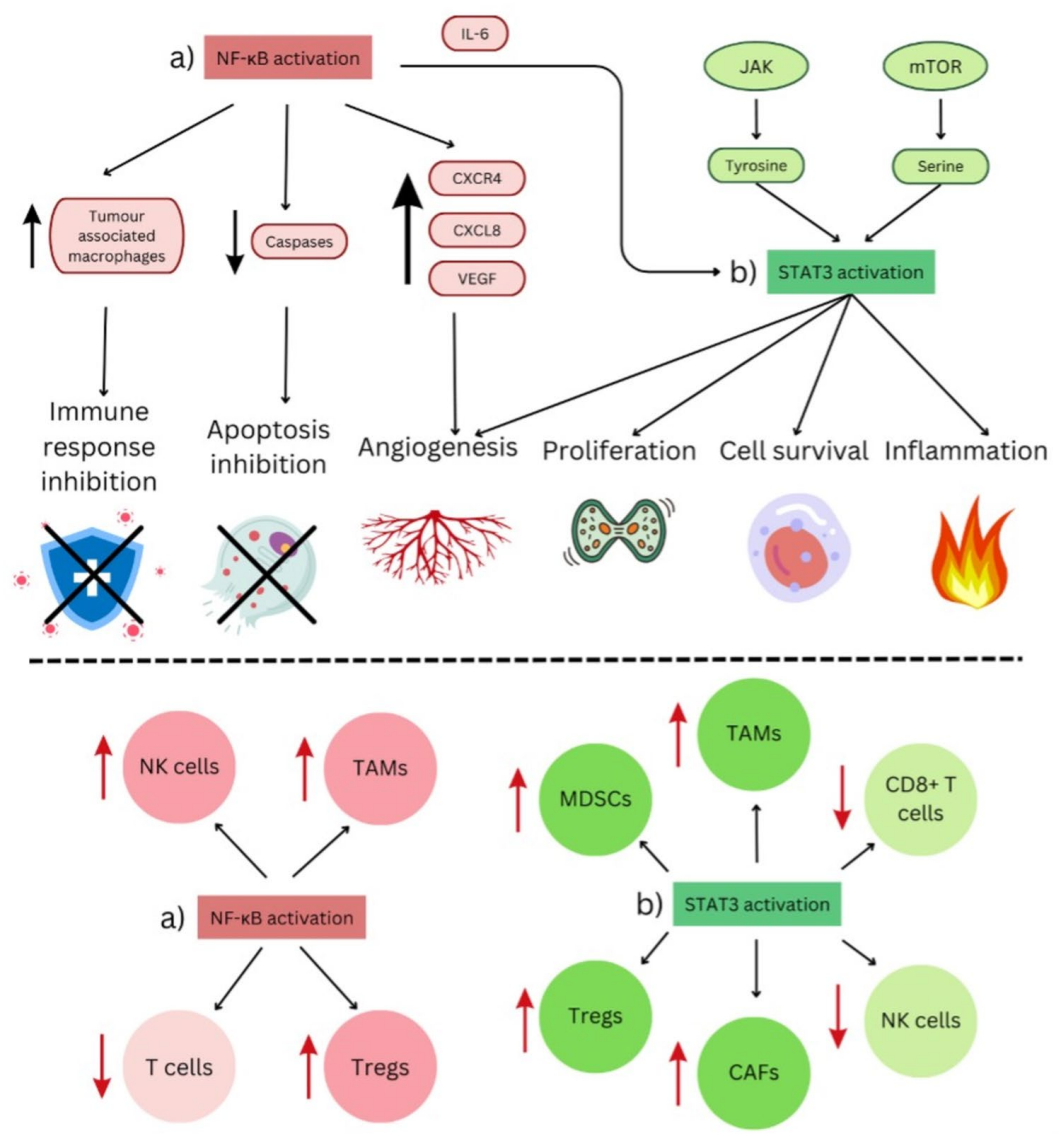
The effects of NF-κB and STAT3 pathways on the lung TME and the cells within

Natural Killer (NK) cells are cytotoxic lymphocytes that kill cancer cells and exhibit strong antitumour effect in many cancers including lung cancer. These cells are predominantly found in the invasive margin surrounding tumour lesions and rarely contacts cancer cells directly. They provide strong protection during the early lung cancer stage, but this effect diminishes as cancer progresses. Defective NK cells in the TME are characterized by reduced toxicity, decreased responsiveness to stimuli and impaired survival [27]. Additionally, defective NK cells and T cell exhaustion are observed in the TME of lung cancer [6]. For example, NK cells may not be able to penetrate the tumour tissues, even with sufficient signalling by chemokines. This has been observed in NSCLC tissues, where NK cells were reported to exist within the stroma that contained active cytokine secretion, but NK cells failed to come into contact and kill tumour cells. The effects of NF-κB and STAT3 pathways on the lung TME and the cells within is illustrated in Fig. 2 and the roles of major immune and stromal cells in the lung cancer TME is tabulated in Table 2 below.

**Table 2:** Roles of major immune and stromal cells in the lung cancer TME

Cell Type	Key Functions & Effects	Reference
TAMs (M1)	• Produce IL-12, IL-23 (antitumour, cytotoxic)	[18]
TAMs (M2)	• Promote stroma formation, angiogenesis, metastasis • Dominant in lung cancer TME	[18]
MDSCs	• Suppress T cells • Secrete VEGF (promotes angiogenesis) • Correlate with poor prognosis	[19, 20]
CD8 + T Cells	• Kill cancer cells • Dysfunctional in advanced lung cancer	[21]
CD4 + T Cells (Tregs)	• Secrete IL-10, TGFβ (immunosuppressive) • Linked to poorer survival • Inhibit effector T cells	[22, 23]
CAFs	• Promote immune evasion, metastasis, angiogenesis • Secrete TDO2 (inhibits dendritic cells)	[24, 25]
NK Cells	• Early antitumour activity • Defective in advanced stages (reduced cytotoxicity)	[6, 28]

### Effect of NF-κB and STAT3 pathways on cellular and molecular components within the lung TME

The NF-κB pathway activates cytokine IL-6, which then interacts with its receptors to subsequently activate the STAT3 signalling pathway. In turn, STAT3 upregulates microRNA-135b (miR-135b), which further activates NF-κB pathway. This crosstalk establishes a positive-feedback loop between the NF-κB and STAT3 pathways, enhancing the progression of NSCLC [28]. The overall impact of the NF-κB and STAT3 pathways on cells within the TME is summarized in the lower half of Fig. 2.

STAT3 is often activated in immune cells infiltrating tumours. In myeloid cells, aberrant STAT3 activation contributes to lung cancer progression by recruiting immunosuppressive cell including regulatory T cells (Tregs), MDSCs and alternatively activated macrophages (M2 macrophage) into the TME. STAT3 regulates M2 macrophages to facilitate tumour growth by promoting immune evasion, supressing immune responses, and enhancing angiogenesis [22]. Tumour-derived gamma-aminobutyric acid (GABA), a neurotransmitter, inhibits M1 macrophage polarization via the JAK2/STAT3 pathways, thereby promoting cancer progression. Additionally, Jumonji Domain-Containing Protein 6 (JMJD6) expressed on the surface of macrophage induces dose-dependent STAT3 phosphorylation, leading to increased expression of the immunosuppressive cytokine IL-10 in TAM through the STAT3 pathway [29].

NF-κB transcription factors also activate TAMs in the tumour stroma to secrete inflammatory cytokines, which suppresses dendritic cells (DCs) maturation and inhibits the adaptive immune response necessary for tumour rejection. When NF-κB is activated by stimuli such as TLR ligands, IL-1β, and TNF-α, it can directly induce macrophage differentiation into M1 phenotype of TAMs, which generally exerts tumour-suppressive effects. However, under different conditions, this activation of NF-κB may also promote the expression of genes that facilitate M2 macrophage polarization, thereby contributing to tumour progression [30]. NF-κB can also be activated by IκKB and has another role of preventing lipopolysaccharide LPS-induced apoptosis in macrophages. Therefore, macrophages deficient in IκKB, have higher sensitivity to LPS-induced apoptosis, and are essentially not effective [12].

STAT3 also plays a crucial role in regulating MDSC, promoting their development and proliferation within tumours. It reduces the production of pro-apoptotic mediators, thereby facilitating apoptosis in cytotoxic T cells that are responsible for eliminating cancers cells. Persistent activation of STAT3 in MDSCs induces the production of immunosuppressive cytokines such as IL-10, TGFβ, and NOX2, which support Treg formation and inhibit dendritic cell (DC) function. Additionally, M2 macrophage and MDSCs secrete cytokines like IL-6 and IL-11, which activate STAT3 and create a signalling loop that fosters a TME favourable for cancer cells.

Research indicates that the expression of polypeptide N-acetyl-galactosaminyltransferase 3 (GALNT3) is diminished in lung cancer tissues compared to normal lung tissues. Low levels of GALNT3 are associated with poorer prognosis in cancer patients. GALNT3 restricts the recruitment of polymorphonuclear myeloid-derived suppressor cells (PMN-MDSCs) by inhibiting the self-renewal of lung cancer cells, which leads to the downregulation of CXCL1. This downregulation is mediated by reduced β-catenin levels, decreased nuclear localization of NF-κB, and diminished c-MET-induced phosphorylation of protein kinase B (AKT) [19]. Therefore, the increased nuclear localization of NF-κB will increase CXCL1 expression and decrease levels of GALNT3 allowing the recruitment of PMN-MDSCs within the lung TME.

Tumour cell-intrinsic STAT3 induces expression of MHC class I, which hinders the cytotoxic activity of NK cells [31]. Besides that, STAT3 also exerts a negative regulatory effect on NK cells activity; its overexpression in NK cells diminishes their cytotoxic function. On the other hand, research directly investigating the role of NF-κB in NK cells within the context of cancer remains limited. One study suggests that within the TME, hypoxia-induced HIF-1α expression inhibits IL-18-mediated NF-κB activation in tumour-infiltrating NK cells. This inhibition resulted in compromised NK cell function and allowed for unchecked tumour growth [32].

In lung cancer, T cells frequently exhibit an exhausted phenotype, characterized by decreased secretion of inflammatory cytokines and reduced cytotoxic activity. STAT3 can attenuate the immune response of T cells by impairing their recruitment, proliferation, and survival. Additionally, STAT3 modulates the expression of immune checkpoint proteins such as PD1 and CTLA4 on T cells, thereby influencing immune tolerance [22]. Conversely, NF-κB is essential for the maturation and functionality of CD8^+^ T cells. In particular, the activation of RelA, which occurs downstream of T cell receptor (TCR) engagement, is crucial for the optimal expression of Eomesodermin (Eomes), a transcription factor required for the development and function of T cells. Moreover, the production of interferon-gamma (IFN-γ) by CD8^+^ T cells rely on NF-κB**,** as evidenced by reduced IFN-γ levels in mice with conditional deletion of RelA in T cells. These findings underscore the significant role of NF-κB in regulating T cell activation, survival, and function, particularly in the context of cancer [32].

STAT3 can enhance the production of Tregs by directly binding to the FOXP3 promoter or by interacting with the promoter of TGFβ and IL-10 in Tregs. Furthermore, STAT3 signalling upregulates CTLA4 expression on Tregs through the IL-10 receptor, thereby augmenting their immunosuppressive ability. On the other hand, excessive activation of NF-κB pathway diminishes T cell function and promotes the activation of Tregs, contributing to immunosuppression. Numerous studies have shown that NF-κB proteins, particularly C-Rel, are vital not only for the transcription of FOXP3 but also for the development of Tregs in the thymus and the function of activated Treg cells (aTregs). The NF-κB pathway also plays a significant role in regulating CTLA4 expression, as well as in the development and maintenance of Treg cells, further contributing to immunosuppression within TME [12].

CAFs are key mediators of immune suppression, extracellular matrix deposition and remodelling, creating a favourable TME that supports cancer progression. The JAK/STAT signalling pathway is constitutively activated in CAFs, and these cells secrete cytokines such as IL-6, IL-10, IL-11, and IL-22, ligands which further amplify the JAK/STAT signalling cascade. Through the IL-6/STAT3 signalling pathway, CAFs enhance the metastatic potential of lung cancer cells, promoting angiogenesis by elevating VEGF and bFGF levels, facilitating cancer invasion and migration. Persistent STAT3 activation in CAFs fosters pro-angiogenic, migratory, and invasive phenotype. Moreover, activation of the JAK/STAT3 signalling cascade can also induce mesenchymal stem cells to differentiate into CAFs within the TME [22]. Besides the role of NF-KB pathway on cancer cells, it is also involved in immune cells such as CAFs and TAMs. It has crucial involvement in CAFs, contributing to their tumour-promoting effects [33]. TLRs are immune cell surface receptors which have been linked to growth of lung cancer cells via anti-apoptotic signals and proliferation [34]. Autophagy mediated HMGB1 is a protein that binds to the TLR4 cell surface receptor which can drive CAFs to affect the NF-κB pathway in lung cancer, contributing to its metastasis [35].

Vascular endothelial growth factor (VEGF) primarily signals through VEGF receptor 2 (VEGFR-2), leading to the activation of STAT3. This activation involves STAT3 dimerization, nuclear translocation, and DNA binding. Once bound to DNA, STAT3 regulates the transcription of genes essential for endothelial cells activation, vascular inflammation, and other related biological processes. A positive-feedback loop is established where VEGF-induced STAT3 activation can directly upregulate the VEGF promoter [36]. Additionally, phosphorylation of JAK2 and subsequent activation of STAT3 can further enhance VEGF/VEGFR-2 signalling, resulting in increased blood vessel permeability. Besides that, NF-κB enhances angiogenesis by upregulating the production of CXCR4 and CXCL8 which are chemokine receptors that subsequently increases VEGFs [12]. The NF-κB and STAT3 pathway interactions in lung cancer TME is tabulated in Table 3 below.

**Table 3:** NF-κB and STAT3 pathway interactions in lung cancer TME

Pathway	Target Cells	Pro-Tumour Effects	Key Mediators	Reference
NF-κB	Macrophages	• M1/M2 polarization balance • Anti-apoptosis (via IκKB) • DC suppression	TLR ligands, IL-1β, TNF-α	[12, 30]
	T cells	• CD8 + T cell maturation • IFN-γ production • Treg development	RelA, Eomes, FOXP3	[32]
	CAFs	• ECM remodelling • Metastasis (via HMGB1/TLR4)	CXCL8, CXCR4	[33, 35]
	Angiogenesis	• VEGF upregulation	CXCL1, β-catenin	[19, 36]
STAT3	Macrophages	• M2 polarization • IL-10 production	GABA, JMJD6	[22, 29]
	MDSCs	• Expansion • Immunosuppression (IL-10/TGFβ)	NOX2	[19, 22]
	T cells	• T cell exhaustion • PD1/CTLA4 upregulation • Treg differentiation	FOXP3, IL-10	[22, 31]
	CAFs	• Pro-angiogenic phenotype • MSC differentiation	VEGF, bFGF	[22]
	NK cells	• Cytotoxicity inhibition • MHC I upregulation	HIF-1α	[31, 32]

### Interaction between cells within the lung TME contributing to tumour development

The interactions between TME components and cells influences tumour aggression and metastasis by enabling cancer cells to acquire an invasive phenotype and spread to distant sites through a metastatic complex [37]. These interactions can also affect tumour growth, angiogenesis as well as immune response and evasion. For example, the interaction of MDSCs with various other cells results in tumour development. MDSCs facilitates the recruitment and formation of Tregs, suppressing the antitumour activity of T cells. Elevated levels of MDSCs with high expression of iNOS and L-arginase I, inhibits the formation of protective CD8^+^ T cell via reduction of CD3ζ chain expression, consequently impairing their immune functionality. Moreover, MDSCs directly induce the expression of T cell exhaustion inhibitory receptors on CD8^+^ T cells, including PD-1, TIGIT, LAG3, CTLA4, and TIM3. For instance, programmed cell death protein 1 (PD-1) interacts with its ligand PD-L1, leading to the inhibition of CD8^+^ T cell proliferation, survival, and cytokine secretion. This interaction impairs immune function, promotes T cells exhaustion, and enhances resistance to immune checkpoint inhibitors (ICIs) thereby facilitating tumour progression [21].

Tregs promote angiogenesis by directly increasing levels of VEGF-A and/or IL-10 or by influencing other immune cells to secrete pro-angiogenic cytokines [38]. Tregs-induced angiogenesis can also be facilitated through the VEGFA/VEGFR2 signalling pathway. Tregs can inhibit the functions of CD8^+^ T cells and dendritic cells via membrane-bound TGF-β, thereby modulating the body’s antitumour immune response. Moreover, Tregs can upregulate the TME by suppressing the secretion of IFN-γ by CD8^+^ T cells, in turn preventing inhibition of sterol regulatory element-binding protein 1 (SREBP1)-dependent fatty acid metabolism which is essential in immunosuppressive M2-(TAMs) survival [39].

In addition, CAFs enhances immune suppression by interacting with M2 macrophages and MDSCs. CAFs inhibits the proliferation of both CD4^+^ and CD8^+^ T cell and reduce the expansion of CD8^+^ T cells within the stimulated CD3^+^ T cells. They also upregulate PD1 protein expression on CD4^+^ and CD8^+^ T cell [40]. Pro-inflammatory cytokines released by CAFs recruit macrophages, neutrophils, and lymphocytes to the tumour stroma. These recruited cells differentiate into TAMs and tumour-associated neutrophils (TANs) which subsequently produces key factors such as VEGF, HGF, MMPs and interleukins, which promotes endothelial growth [41].

## Therapeutic implications

### STAT3

STAT3 is an important target in the therapy of lung cancer as it plays various crucial roles in tumour development including tumour cell proliferation, tumour cell survival and evasion of pathogen from the immune system. It is primarily activated by two major pathways which are the mTOR and JAK pathways. The inhibition of both these pathways provides an effective strategy in overcoming the activation of the STAT3, which would directly inhibit the development and progression of lung cancer.

In the case of the mTOR pathway, its inhibition has emerged as a promising therapeutic strategy by promoting apoptosis in cancer cells and overcoming resistance towards treatments. This is because mTOR can activate the STAT3 pathway and together, the mTOR/STAT3 pathway promotes the development of tumours [42]. Hence, therapeutic strategies that focus on inhibiting the mTOR pathway, which subsequently inhibits the STAT3 activation, need to be implemented.

There are several mTOR inhibitors being studied and developed to have enhanced selectivity and robust anti-cancer effects. Amongst these would be the dual-action pharmacological agents that can modulate two targets simultaneously, such as CC-115 which suppresses both mTORC1 and mTORC2 pathways along with the DNA-dependent protein kinase (DNA-PK), which inhibits cancer cell growth in vivo and in vitro [43].

Additionally, mTOR also regulates the programmed death ligand 1 (PD-L1) expression, which allows for cancer cells to evade or inhibit the immune system. Therefore, with the combination of mTOR inhibitors and ICIs such as PD-1/PD-L1 blockers, research shows that therapeutic efficacy is elevated, which has potential to overcome the development of resistance [44]. For example, the mTOR inhibitor vistusertib combined with ICIs such as αPD-1, αCTLA-4 and αPD-L1, produced synergistic anti-cancer effects compared to when these agents were administered individually [45].

Moreover, researchers have proposed new immune checkpoint molecules, such as TIM-3, LAG-3 and TIGIT, to overcome resistance of the cancer cells to ICIs [6]. These molecules play a role in the modulation of multiple aspects of the immune response, resulting in a versatile attack on the tumour. There is also an improvement in antitumour immunity when mTOR inhibitors are combined with these immune checkpoint molecules.

Furthermore, transcription factors are responsible for the mediation of the mTOR signalling pathway, causing gene expression reprogramming, which ultimately promotes the growth of tumour as well as its survival. So, to inhibit the mTOR pathway to avoid activation of STAT3, the transcription factors must be targeted, and to do so, small molecule inhibitors must be deployed. For instance, studies have shown that PP242 can inhibit both the mTORC1/2 pathway [46]. Thus, targeting the transcription factors involved may be an effective method to avoid the activation of STAT3 pathway.

**Fig. 3:**
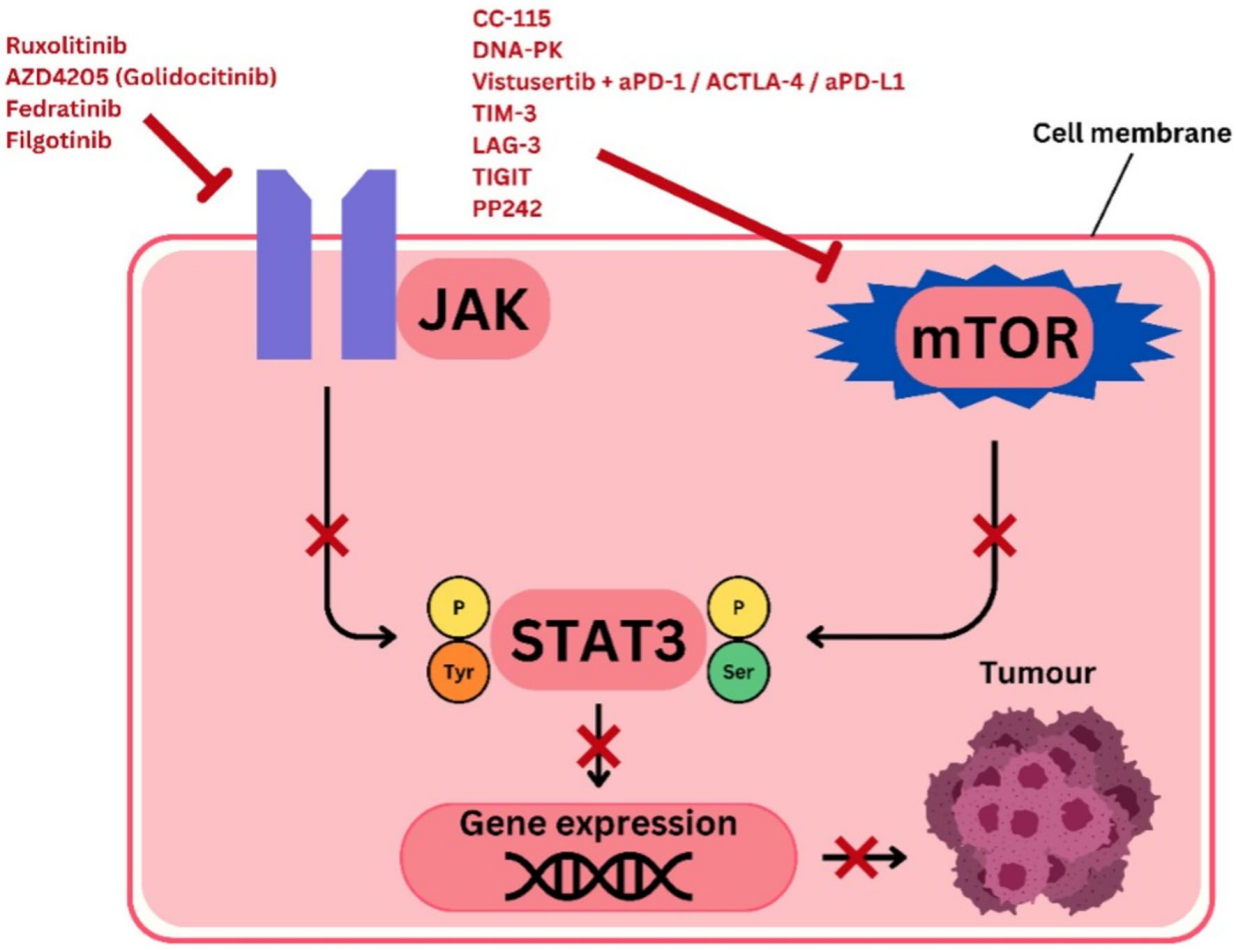
Apoptosis promoting properties of the inhibitors such as ruxolitinib, AZD4205 (Golidocitinib), fedratinib and filgotinib, supresses the activation of STAT3 by inhibiting the phosphorylation of tyrosine by JAK, whereas CC-115, DNA-PK, TIM-3, LAG-3, TIGIT PP242 and the combination of vistusertib with aPD-1, ACTLA-4 or aPD-L1 inhibits the activation of STAT3 by supressing the phosphorylation of serine by JAK. The inactivation of the STAT3 pathway inhibits its ability to drive tumour formation via gene expression, hence ultimately inhibiting the growth, survival, and proliferation of tumour cells

The JAK pathway also could activate STAT3. Hence, multiple measures can be taken to inhibit the JAK pathway by extension disrupts the STAT3 pathway, which plays a critical role in lung TME. These inhibitors work by targeting different JAK family members, as shown in Fig. 3, thus interfering with THE STAT3 pathway which is a crucial signalling pathway that promote cancer cell survival and proliferation through gene expression. For instance, Ruxolitinib, an inhibitor of JAK1 and JAK2, competitively inhibits the ATP-binding catalytic site on these kinases [47]. A Phase Ib study combining ruxolitinib with afatinib, an EGFR inhibitor, in NSCLC patients demonstrated that this regiment was well-tolerated and displayed activity against the malignancy, with 23.3% showing partial responses and 80% having stable disease [48]. However, despite these promising results, many trials with ruxolitinib have been underperforming, potentially due to its impact on immune cell function, which might counteract its anti-cancer effects.

Moreover, AZD4205 (Golidocitinib), a selective JAK1 inhibitor, has also shown significant pre-clinical efficacy [49]. In a pre-clinical NSCLC in vivo model, AZD4205 treatment inhibited both tumour growth and STAT3 activation [50]. These effects were more pronounced when AZD4205 was used in combination with osimertinib, another EGFR inhibitor [51]. This combination has progressed to Phase I/II clinical trials, highlighting its potential therapeutic value [50]. On the other hand, Fedratinib targets JAK2 and has been found to be effective in NSCLC cells with elevated JAK2 expression [52]. In combination with erlotinib, an EGFR tyrosine kinase inhibitor, fedratinib decreased STAT3 activation and increased apoptosis in erlotinib-resistant NSCLC cells, also inhibiting tumour growth in murine models [53].

Furthermore, Filgotinib, a selective JAK1 inhibitor, reduces levels of cytokines such as IL-6 and inhibits STAT3 activation in NSCLC cells [54]. Filgotinib demonstrates similar efficacy in inhibiting JAK1-mediated IFNα, and IL-6 responses compared to other JAK inhibitors [55]. In NCI-H889 lung cancer cells, derived from a metastatic site, Filgotinib effectively inhibited STAT3 activation, underscoring its potential in targeting the TME [56]. In short, the mTOR and JAK pathways affects the activation of STAT3, which leads to tumour development and progression. Hence, necessary measures need to be taken in order to inhibit the activation of STAT3 at its roots.

### NFKB

Inhibitors of the NF-κB pathway have significant therapeutic implications for lung tumours by disrupting key signalling mechanisms that contribute to tumour growth and survival. For instance, Siltuximab is an IL-6 inhibitor that fully eliminates IL-6 signal transduction in vivo and in vitro, effectively inhibiting lung cancer proliferation [57]. In a phase I/II clinical trial evaluating its efficacy in solid tumours, including pancreatic cancer, siltuximab was well tolerated but showed no significant clinical activity, with adverse events such as abnormal liver function and fatigue occurring in more than 10% of patients [58]. Despite promising in vitro and in vivo results, further research is needed to improve clinical outcomes for cancer patients [59].

Additionally, Bortezomib, a proteasome inhibitor, indirectly targets the NF-κB pathway by interfering with the degradation of key regulatory proteins [60]. Adding bortezomib to standard chemotherapy regimens holds promise for enhancing treatment efficacy, especially in advanced stages of SCLC. In one study conducted both pre-clinical and clinical studies, bortezomib has shown significant therapeutic benefits in multiple myeloma (MM) [56]. Bone marrow stromal cells (BMSCs) can upregulate IL-6 expression via NF-κB –dependent mechanisms, suggesting that inhibiting NF-κB could reduce IL-6 levels and prevent the triggering of terminal differentiation. Research indicates that bortezomib can downregulate NF-κB target genes in both pre-clinical and clinical settings, highlighting its potential in cancer therapy [61]. Moreover, bortezomib induces cell cycle arrest at G2/M phase transition in replicating cells in vitro, disrupting cell cycle progression and leading to apoptosis [62]. This effect is partly due to the inhibition of the NF-κB pathway. Additionally, bortezomib reduces VEGF secretion within the bone marrow microenvironment, inhibiting the activation of caveolin-1, a protein crucial for motility of a cell and angiogenesis. This inhibition of VEGF-triggered tyrosine phosphorylation and subsequent angiogenetic processes has been validated through in vivo experimentation [63]. Clinical trials have further supported the use of bortezomib in combination with pemetrexed, demonstrating that this combination is both tolerable and effective for patients with advanced solid tumours, including NSCLC [64].

Furthermore, Carfilzomib is an irreversible second-generation proteosome inhibitor that has shown promising results in cancer treatment [65]. In multiple myeloma (MM) cells exposed to carfilzomib, both the extrinsic and intrinsic apoptotic pathways are activated, leading to significant increases in caspases-3,-7,-8, and –9 [61]. Caspase-3 and caspase-7 are critical effector caspases that exist as inactive zymogens and once activated by initiators, inactivate various structural and functional proteins, culminating in apoptosis [66]. Caspase-8 initiates the death receptor pathway, whilst caspase-9 initiates the mitochondrial pathway [66]. Both caspases are located upstream in their respective pathways and are essential for transmitting and amplifying apoptotic signals, thereby activating downstream effector genes that induce apoptosis [66]. Compared to bortezomib, carfilzomib exhibits minimal off-target activity beyond the proteasome, enabling it to induce apoptosis in both bortezomib-naive and bortezomib pre-treated MM cells without increasing toxicity [56]. This specificity, along with its higher affinity for the proteosome, contributes to carfilzomib’s superior efficacy in xenograft models [61]. These attributes suggest that carfilzomib may offer a potent antitumour response with fewer side effects compared to bortezomib, highlighting its potential as a more effective treatment option for patients with MM [67].

**Fig. 4:**
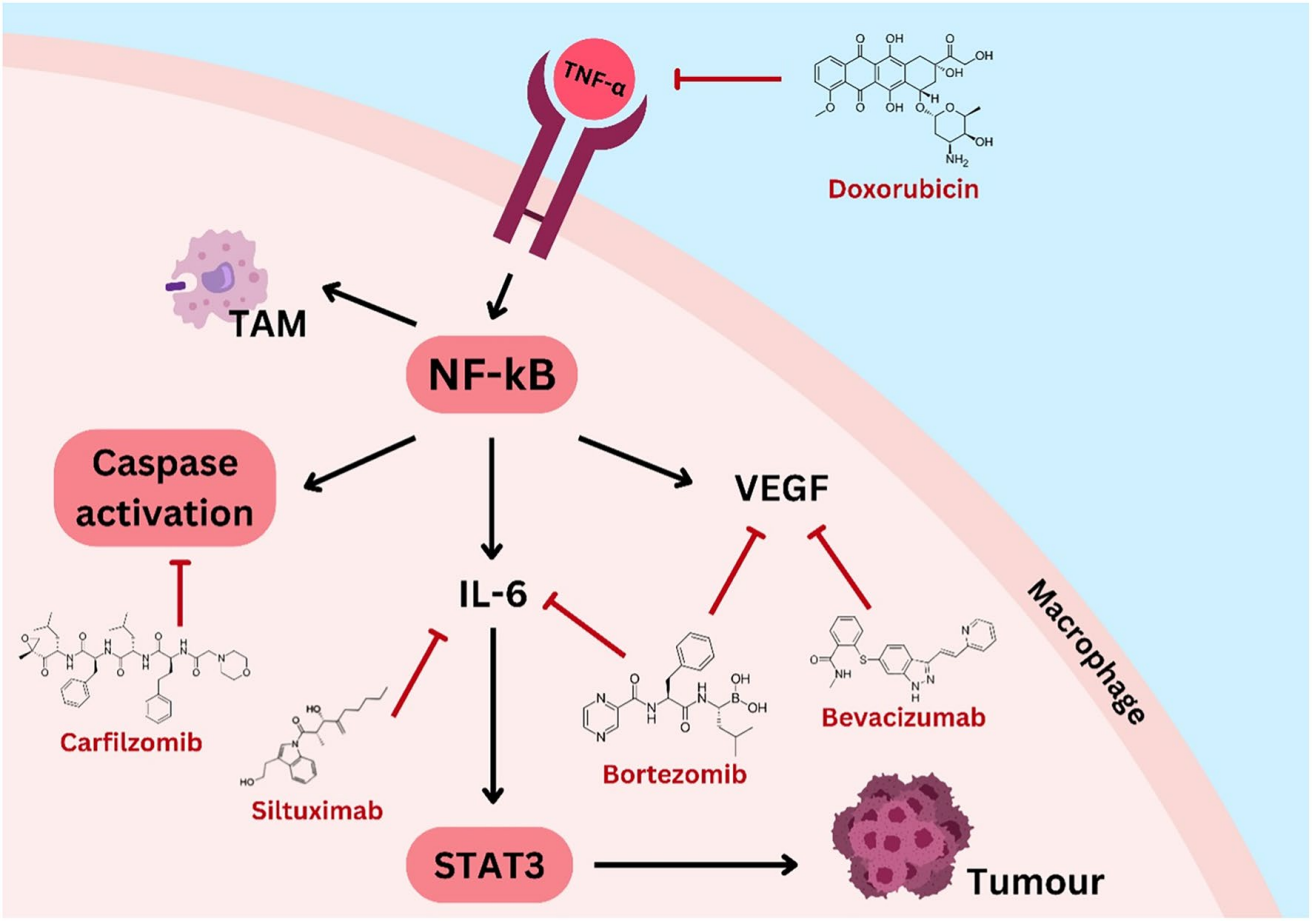
The NF-κB pathway in a macrophage is activated by TNF-α, which leads to tumour formation. To inhibit TNF- α binding, doxorubicin can be deployed. Secondly, carfilzomib induces caspase activation and Siltuximab which inhibits IL-6 are treatments that trailed to be administer to patients. Fourthly, Bortezomib targets both NF-κB via IL-6 and VEGF. Lastly, Bevacizumab is shown to decrease VEGF levels to counter tumour proliferation. The pathway also highlights the role of TAMs in the TME and the downstream effects of STAT3 activation, promoting tumour growth and immune evasion

Besides that, Doxorubicin Hydrochloride works by inducing phosphorylation and degradation in IκB, leading to the inhibition of NF-κB in A549 human lung adenocarcinoma cell lines, as shown in Fig. 4 [49]. When combined with sulfasalazine, doxorubicin’s cytotoxicity is enhanced, inhibiting TNF-α in NF-κB activation [49]. On the other hand, Bevacizumab is a humanised monoclonal antibody against VEGF, used in combination with standard chemotherapeutics for treating stage IIIb and IV NSCLC [68]. Bevacizumab decreases NF-κB levels, countering the increase induced by treatments like TBi, which helps in reducing tumour proliferation and improving patient outcomes [69].

### Other relevant factors affecting TME

There are several therapeutic implications of several other factors that also affect the TME, causing progression of cancer. For instance, the immunosuppressive cells MDSC which stimulates the growth of tumour by the suppression of CD4^+^ and CD8^+^ T cells, which are essential immune cells that have antitumour properties. CD8^+^ recognises tumour cells sign their MHC class-I receptor and causes the tumour cells to lyse whereas CD4^+^ can be differentiated into several T helper (Th) cells and Treg cells that secrete cytokines to protect the host from pathogens [70]. Inhibition of such vital cells causes the proliferation and growth of tumour cells. Hence, to modulate the activity of MDSC, measures need to be taken to reduce its ability to suppress the T cells. For example, bacterial strains, such as *Klebsiella pneumoniae* and *Pseudomonas aeruginosa*, have been shown to produce necessary metabolites that are able to suppress the function of MDSC, thereby avoiding the CD4^+^ and CD8^+^ T cells suppression, which ultimately enhances the antitumour immunity [71].

Besides that, to enhance the function and activity of CD8^+^ T cells, probiotics and prebiotics can be combined and used with pre-existing immunotherapies to improve the therapeutic outcomes. Example of probiotics includes *lactobacillus rhamnosus*, which stimulates dendritic cells through the Toll-like receptor 2 (TLR2) pathway hence activating the CD8^+^ T cells, as well as *Eubacterium hallii*, which promotes the activation of CD8^+^ T cells and allows them to easily infiltrate into the tumours [72, 73]. On the other hand, prebiotics such as inulin triggers the T cells activation, which leads to an increase in the number of CD8^+^ T cells produced [74].

Additionally, the Treg cell, which is the differentiated naive CD4^+^ T cells, can inhibit the immune responses that are excessive or uncontrolled, leading to the suppression of immune cells such as T cells, B cells and natural killer (NK) cells [75]. Therefore, the microbiome should be modulated to reduce the activity of Treg cells. Just like MDSC, Treg cells can be modulated using bile acid metabolite in bacteria from the Bacteroidetes species, isoallolithocholic acid (isoalloLCA), which has the potential to promote the differentiation of Treg cells [76].

Another factor that affects TME is the cells that detect and eradicate tumour cells in the preliminary stages of lung cancer by playing a vital role in the innate immune response system, which are the NK cells [77]. To enhance the potency of NK cells against tumour cells, there is a strategy known as the chemical antigen receptor (CAR) technology. Originally, this technology was used to produce T cells but now it has been adapted to produce CAR-NK cells using the same domain used to produce CAR-T cells [78].

Furthermore, TAMs are macrophages that can polarise into pro-tumoural or antitumoural phenotypes depending on TME. Several bacterial products can polarise TAMs into the antitumoural phenotype, which allows them to lyse tumour cells [79]. Therefore, more research needs to be conducted to detect which bacterial products affect the polarisation and to determine if the bacteria could be safely introduced to the body to increase the potency of TAMs.

Other than that, CAFs have the ability to promote tumours by stimulating growth and metastasis. Hence, CAFs have to be inhibited, and this is possible by using clodronate-containing liposomes (Clo-Lipo-DOTAP) to clear TAMs which significantly suppresses the development of tumour [80]. On the other hand, VEGF plays a significant role in angiogenesis by providing blood supply to tumours and allowing them to grow. It does this by targeting the endothelial cells of blood vessels which triggers an intracellular signalling pathway that leads to the formation of new blood vessels. Therefore, to inhibit the VEGF signalling pathways that cause angiogenesis, there are several FDA approved drugs that can be used, such as Bevacizumab, which has potent inhibitory ability [81].

In short, studying and understanding the modulation of the TME and the relevant factors contributing to its balance, as listed in Table 4, has the potential to offer new directions especially in improving the efficacy of current cancer treatments. Further research into the microbial interactions and their effects on immune cells holds immense potential significantly in the development of new therapies aiming to help patients suffering from lung cancer.

**Table 4:** Overview of Therapeutic Agents Targeting TME Pathways

Target	Therapeutic Approach	Key Agents	Mechanism/Outcome	Reference
STAT3	mTOR pathway inhibition	CC-115, PP242, Vistusertib + ICIs (αPD-1/αPD-L1/αCTLA-4)	Blocks STAT3 activation; enhances apoptosis and combats ICI resistance	[42–46]
	JAK pathway inhibition	Ruxolitinib (JAK1/2), AZD4205 (JAK1), Fedratinib (JAK2), Filgotinib (JAK1)	Reduces STAT3 phosphorylation; inhibits tumour growth (synergy with EGFR inhibitors)	[47–56]
NF-κB	IL-6 inhibition	Siltuximab	Suppresses IL-6 signalling; inhibits proliferation (limited clinical efficacy)	[57–59]
	Proteasome inhibition	Bortezomib, Carfilzomib	Induces apoptosis via caspase activation; reduces VEGF/IL-6 (superior efficacy with carfilzomib)	[60–67]
	IκB degradation/VEGF blockade	Doxorubicin + Sulfasalazine, Bevacizumab	Inhibits NF-κB activation; counters angiogenesis	[49, 68, 69]
Other TME Factors	MDSC modulation	Bacterial metabolites (Klebsiella/Pseudomonas)	Suppresses MDSC function; restores CD4 + /CD8 + T cell activity	[70, 71]
	CD8 + T cell enhancement	Probiotics (Lactobacillus rhamnosus), Prebiotics (Inulin)	Activates dendritic cells (TLR2) and boosts T cell infiltration	[72–74]
	Treg modulation	Bacteroidetes-derived isoalloLCA	Promotes Treg differentiation; balances immune response	[75, 76]
	NK cell enhancement	CAR-NK cell therapy	Improves tumour cell targeting via engineered receptors	[77, 78]
	TAM reprogramming	Bacterial products	Polarizes TAMs to antitumoural phenotype	[79]
	CAF inhibition	Clo-Lipo-DOTAP	Clears TAMs; suppresses tumour growth	[80]
	Angiogenesis inhibition	Bevacizumab	Blocks VEGF signalling; reduces blood vessel formation	(81)

## Conclusion

In conclusion, the growth and progression of lung cancer are significantly influenced by the lung TME. The interaction and effects of the complex networks of cellular and non-cellular components, signalling molecules and extracellular matrix components enables cancer cells to proliferate, metastasize and evade the immune system. Therefore, it is crucial to target the components involved in the lung TME in cancer therapy due to their ability of creating a milieu that is favourable for tumour maturation and development.

The pro-tumourigenic status of the TME is mainly dependent on two key signalling pathways, namely the NF-κB and STAT3 pathways. The activation of these two pathways leads to the upregulation and downregulation of the various cellular and non-cellular components in the TME, ultimately resulting in immune evasion, inhibition of apoptosis, angiogenesis, tumour cell proliferation, survival, and inflammation. Therefore, inhibiting the activation of these pathways represents a promising therapeutic strategy. Pre-clinical and clinical research have demonstrated the potential of targeting these pathways with inhibitors, such as mTOR and JAK inhibitors for the STAT3 pathway along with a few other inhibitors such as the IL-6 and VEGF inhibitors for the NF-κB pathway. These therapeutic strategies can help to improve patient outcomes and perhaps overcome resistance to current treatments.

Overall, understanding the effects of the functions and interactions of the various cellular and non-cellular components in the TME such as regulatory T cells (Tregs), myeloid-derived suppressor cells (MDSCs), tumour-associated macrophages (TAMs), CAF, natural killer cells (NK), T cells and VEGF, offers additional insights into developing tailored therapeutic interventions. The interplay between these immune cells and the major pathways involved (NF-κB and STAT3) suggests that disrupting the TME and suppressing cancer progression may require a multimodal approach that involves immunomodulatory drugs as well as pathway inhibitors. For this reason, future research should focus on improving therapeutic efficacy and addressing resistance in lung cancer treatment.

## Data Availability

No datasets were generated or analyzed during the current study.

## Abbreviations

AKT: Protein kinase B
CAFs: Cancer-associated fibroblasts
CD4^+^: Cluster of differentiation 4 positive
CD8^+^: Cluster of differentiation 8 positive
CTLA4: Cytotoxic T-lymphocyte associated protein 4
CXCR4: C-X-C motif chemokine receptor 4
CXCL8: C-X-C motif chemokine ligand 8
DC: Dendritic Cells
ECM: Extracellular matrix
FOXP3: Forkhead box P3
GABA: Gamma-A minobutyric acid
GALNT3: Polypeptide N-acetyl-galactosaminyl transferase 3
HGF: Hepatocyte growth factor
HIF: Hypoxia-inducible factor
HIF-1α: Hypoxia-inducible factor-1 α
HMGB1: High mobility group box 1
IAPs: Inhibitors of apoptosis
ICIs: Immune checkpoint inhibitors
IFN-γ: Interferon-gamma
IKK: Inhibitory kappa B kinase
IL: Interleukin
iNOS: Inducible nitric oxide synthase
JAK: Janus kinase
JMJD6: Jumonji domain-containing protein 6
LAG3: Lymphocyte-activation gene 3
LPS: Lipopolysaccharide
MDSCs: Myeloid-derived suppressor cells
miR-135b: MicroRNA-135b
MMPs: Matrix metalloproteinases
mTORC1: Mechanistic target of rapamycin complex 1
myCAFs: Myofibroblastic CAFs
NF-κB: Nuclear factor kappa B
NK: Natural killer
PD1: Programmed cell death protein 1
PD-L1: Programmed death ligand 1
PMN-MDSCs: Polymorphonuclear myeloid-derived suppressor cells
NOX2: NADPH oxidase 2
NSCLC: Non-small cell lung cancer
SCLC: Small cell lung cancer
SREBP1: Sterol regulatory element-binding protein 1
SRGN: Serglycin
STAT3: Signal transducer and activator of transcription 3
TAMs: Tumour-associated macrophages
TANs: Tumour-associated neutrophils
TCR: T cell receptor
TDO2: Tryptophan 2–3-dioxygenase
TGFβ: Transforming growth factor β
TIGIT: T cell immunoreceptor with Ig and ITIM domains
TIM3: T cell immunoglobulin and mucin-domain containing-3
TNF-α: Tumour necrosis factor alpha
Th2: T helper 2
TLR: Toll-like receptor
TME: Tumour microenvironment
Tregs: Regulatory T cells
VEGF: Vascular endothelial growth factor
